# Preconception maternal nutrition: a multi-site randomized controlled trial

**DOI:** 10.1186/1471-2393-14-111

**Published:** 2014-03-20

**Authors:** K Michael Hambidge, Nancy F Krebs, Jamie E Westcott, Ana Garces, Shivaprasad S Goudar, Balachandra S Kodkany, Omrana Pasha, Antoinette Tshefu, Carl L Bose, Lester Figueroa, Robert L Goldenberg, Richard J Derman, Jacob E Friedman, Daniel N Frank, Elizabeth M McClure, Kristen Stolka, Abhik Das, Marion Koso-Thomas, Shelly Sundberg

**Affiliations:** 1 University of Colorado Denver, Aurora, CO, USA; 2 Francisco Marroquin University, Guatemala City, Guatemala; 3 KLE University’s Jawaharlal Nehru Medical College, Belgaum, Karnataka, India; 4 Aga Khan University, Karachi, Pakistan; 5 Kinshasa School of Public Health, Kinshasa, Democratic Republic of Congo (DRC; 6 University of North Carolina, Chapel Hill, NC, USA; 7 FANCAP, Guatemala City, Guatemala; 8 Columbia University, New York, NY, USA; 9 Christiana Care, Newark, DE, USA; 10 RTI International, Research Triangle Park, NC, USA; 11 Eunice Kennedy Shriver National Institute of Child Health and Human Development, Rockville, MD, USA; 12 Bill & Melinda Gates Foundation, Seattle, WA, USA

**Keywords:** Preconception, Maternal, Nutrition, Birth length, Epigenetics, Microbiome

## Abstract

**Background:**

Research directed to optimizing maternal nutrition commencing prior to conception remains very limited, despite suggestive evidence of its importance in addition to ensuring an optimal nutrition environment in the periconceptional period and throughout the first trimester of pregnancy.

**Methods/Study design:**

This is an individually randomized controlled trial of the impact on birth length (primary outcome) of the time at which a maternal nutrition intervention is commenced: Arm 1: ≥ 3 mo preconception vs. Arm 2: 12-14 wk gestation vs. Arm 3: none.

192 (derived from 480) randomized mothers and living offspring in each arm in each of four research sites (Guatemala, India, Pakistan, Democratic Republic of the Congo). The intervention is a daily 20 g lipid-based (118 kcal) multi-micronutient (MMN) supplement. Women randomized to receive this intervention with body mass index (BMI) <20 or whose gestational weight gain is low will receive an additional 300 kcal/d as a balanced energy-protein supplement. Researchers will visit homes biweekly to deliver intervention and monitor compliance, pregnancy status and morbidity; ensure prenatal and delivery care; and promote breast feeding. The primary outcome is birth length. Secondary outcomes include: fetal length at 12 and 34 wk; incidence of low birth weight (LBW); neonatal/infant anthropometry 0-6 mo of age; infectious disease morbidity; maternal, fetal, newborn, and infant epigenetics; maternal and infant nutritional status; maternal and infant microbiome; gut inflammatory biomarkers and bioactive and nutritive compounds in breast milk. The primary analysis will compare birth Length-for-Age Z-score (LAZ) among trial arms (independently for each site, estimated effect size: 0.35). Additional statistical analyses will examine the secondary outcomes and a pooled analysis of data from all sites.

**Discussion:**

Positive results of this trial will support a paradigm shift in attention to nutrition of all females of child-bearing age.

**Trial registration:**

ClinicalTrials.gov NCT01883193.

## Background

Poor nutrition during pregnancy is a significant contributor to maternal morbidity and a leading cause of childhood mortality and morbidity worldwide. There is universal recognition of the importance of the ‘The First Thousand Days’ for physical growth and, when linear growth fails during this ‘window of opportunity’, for associated concurrent and future morbidity and mortality [1-3]. Sub-optimal nutrition is widely regarded as the most important etiologic factor contributing to impaired linear growth in poor communities worldwide. However, multiple nutrition interventions, whether maternal following identification of pregnancy, or offspring < 2 years of age have overall met with only partial success [4]. Typically, however, maternal intervention trials have not begun in the first trimester or/and the periconceptional period despite observational evidence of a relationship between poor maternal nutrition at conception and LBW first reported a quarter of a century ago [5]. A series of observational and experimental studies suggest that intra-uterine growth retardation (IUGR), preterm birth (PTB) and stillbirths have their origins in part in early pregnancy [6-8]. When the fetus is smaller than expected in the first trimester, there is increased prevalence of PTB and IUGR [7,8]. Low maternal weight gain in early pregnancy, indicative of sub-optimal maternal nutrition, has been reported to be a specific cause of LBW attributable to effects on both length of gestation and on fetal growth velocity [9]. An intervention improving maternal nutritional status and maternal weight in early pregnancy was associated with a positive effect on birth length which was not observed with nutrition interventions starting later in pregnancy [9]. The results of a recent intervention study in Bangladesh [10] have demonstrated the importance of the time of gestation at which a nutrition intervention is commenced. When the intervention was started in the first trimester (average of 9 wk gestational age), the beneficial effects on birth weight and even on offspring mortality were significantly greater than when the same intervention was commenced at 20 wk. Maternal-infant bonding and perception of food insecurity also benefited from the first trimester start [11].

Studies with animal models have demonstrated that fetal growth and development are most vulnerable to maternal nutrition deficiencies in early gestation, specifically during the implantation period and that of rapid fetal development [12]. A similar conclusion has been based on analyses of observational studies in the human with the emphasis on the need for well-designed periconceptional intervention trials in developing country settings [13]. Concerns that maternal short stature resulting from intergenerational effects and the maternal environment during her own ‘window of opportunity’ may severely limit the benefits of maternal nutrition intervention have recently been modified [14], in part on the basis of rapid enhancement in linear growth over one generation or less when there are major environmental improvements [15]. To be certain that the effects of any preconception intervention will be achieved by the time the ovum is fertilized, the interval between starting the intervention and conception must be adequate. Extrapolation from experimental data from dietary-restricted sheep [16] suggests that such interventions should start ≥ 3 mo prior to conception. Limited human data indicate that the greatest effects of maternal nutrition supplements on birth weight have occurred when maternal nutritional status is poor and when supplements were continued from the previous pregnancy until the next [17].

Selection of the correct primary outcome for a preconception maternal nutrition trial is critical. A special feature of this project is the selection of birth length (length-for-age Z-score, LAZ) as the primary outcome. There are two reasons for this choice. First is the evidence that birth length, in contrast to birth weight, is determined primarily by development early in fetal life [8]. A nutrition intervention for underweight women was associated with a positive effect on birth length which was not observed when the intervention was started later in pregnancy [9]. These results have similarities to the results of the study in Burkina Faso in which supplements had an effect on birth weight but not on birth length except on underweight women [18]. Birth LAZ is the strongest predictor of LAZ at least until mid-infancy [19], while weight for age z-scores (WAZ) at birth have no predictive value of length in mid-infancy. This is important because it is retarded linear growth rather than wasting in the first two years that has the strongest association with infant/young child morbidity/mortality and with non-communicable disease later in life.

Increasingly, the argument for commencing maternal nutrition trials prior to conception is being strengthened by epigenetic studies. For example, in non-human primates, manipulation of the diet for relatively short periods prior to conception can alter the maternal and fetal epigenome with corresponding fetal changes in the metabolic phenotype [20-23]. In the human, periconceptional micronutrient supplementation favorably impacts methylation patterns in cord blood and in the young infant [24]. The closer to fertilization, the greater the potential for epigenetic changes and corresponding plasticity of the offspring in response to environmental change. These changes in the placenta and embryo/fetus provide a very plausible explanation for the concept of the fetal origins of adult disease [16]. Though these epigenetic changes are potentially reversible, little is yet known about the speed and extent of these improvements in response to improvements in the environment, including the nutrition environment, nor is it yet known how dependent these are on the prior duration of the effects of a poor maternal environment. Epigenetic studies are sorely needed in this area.

Maternal nutrition has received relatively little attention in the context of implementing the United Nations’ Millennium Development Goals (MDG) [25]. This can be attributed to the lack of a strong evidence base to justify the huge effort necessary to optimize the nutrition status of virtually all females of reproductive age. Rigorous multinational trials demonstrating the efficacy of preconception nutrition in improving offspring outcomes are a critical need.

### Objectives and hypotheses

#### Goal

The goal of this trial is to ascertain the benefits to the offspring of ensuring optimal human maternal nutrition before conception, during the peri-conceptional period and during the entire first trimester.

#### Objective

The objective is to determine the benefits to the offspring of women in poor, food-insecure environments of commencing a daily comprehensive maternal nutrition supplement (with additional balanced calorie/protein supplement for underweight participants) ≥ 3 mo prior to conception versus the benefits of commencing the same supplement at 12-14 wk gestation and also to compare offspring outcomes with those of a third trial arm who receive no supplement.

#### Primary hypothesis

In women living in poor, food insecure populations, commencing a maternal nutrition supplement at least 3 mo prior to pregnancy (Arm 1) will result in significantly greater fetal linear growth as determined by newborn LAZ than starting the same nutrition supplement at 12-14 wk gestation (Arm 2) or than not providing this supplement (Arm 3).

#### Secondary hypotheses

Commencing the nutrition intervention prior to conception will also be associated with:

a. Greater fetal linear growth rate which will be maintained at least until mid-infancy (6 mo postpartum);

b. Higher mean birth weight than if the same supplement is delayed until 12 wk gestation (Arm 2) or not provided (Arm 3);

c. Decrease in hospital/health center visits/admissions for sepsis/respiratory disease and diarrhea (and of calendar recorded morbidity at home);

d. Positive changes in the maternal, placental and offspring epigenome which may be related to growth and development of the offspring;

e. Distinct differences in the gut and vaginal microbiota of the mother and gut of the infant;

f. Improvement of maternal and offspring inflammation and favorable changes in the nutritional and bioactive components of breast milk at 2 wk and 3 mo postpartum.

### Site description

Rural sites in four countries will participate in a common trial format, with each site powered independently for the primary outcome, birth length. The sites are in India (Belgaum, Karnataka), Pakistan (Thatta, Sind Province), Democratic Republic of Congo (DRC, Equateur Province), and Guatemala (Chimaltenango Department), all of which have a 10-year history of working together primarily on shared common trials through the *Eunice Kennedy Shriver* National Institute for Child Health and Human Development (NICHD) Global Network (GN) for Women’s and Children’s Health Research (http://gn.rti.org).

### Trial preparation

Preparatory measures have included the formation of a Scientific Advisory Committee (membership details provided in Additional file 1) which has been active in providing key advice reflected in the final trial protocol, including the composition of the nutrition interventions.

Another special measure has been to establish the use of data from the GN Maternal Newborn Health (MNH) Registry [26] or/and other strategies including home surveys to assess site specific inter-pregnancy intervals and optimal parity to achieve 50% conception within the timeline of the trial.

Training is being undertaken by the University of Colorado (UCD) team and RTI, the Data Coordinating Center for the GN and this trial, in both train-the-trainer meetings of all site leaders and by on-site training at each of the four sites.

A common manual of operations and data collection tools have been developed by UCD and RTI and translated by all four sites.

Relevant pilot infant linear growth data have been provided by two very recent studies [27,28]. Newborn length data are available only for Guatemala where the mean Z-score in Chimaltenango Department for a recent cohort of 148 subjects was −1.0 [19]. Mean (SD) birth weights derived from recent GN MNH Registry data are: India 2759 (435) g; Pakistan 2974 (522) g; DRC 3134 (478) g; Guatemala 3018 (478) g.

Other preparatory measures include:

• Ethics committee and local Ministries of Health approval at each research site and IRB approval at University of Colorado;

• Development/strengthening of links with the local health services and regional hospitals;

• Contractual arrangements for awards and subcontracts;

• Community, including civil and local health facility, approval;

• Development of data collection forms and preparation of manual of operations;

• Preparation, procurement, analyses, shipment and import of supplements.

## Methods

### Study design

**Figure 1:**
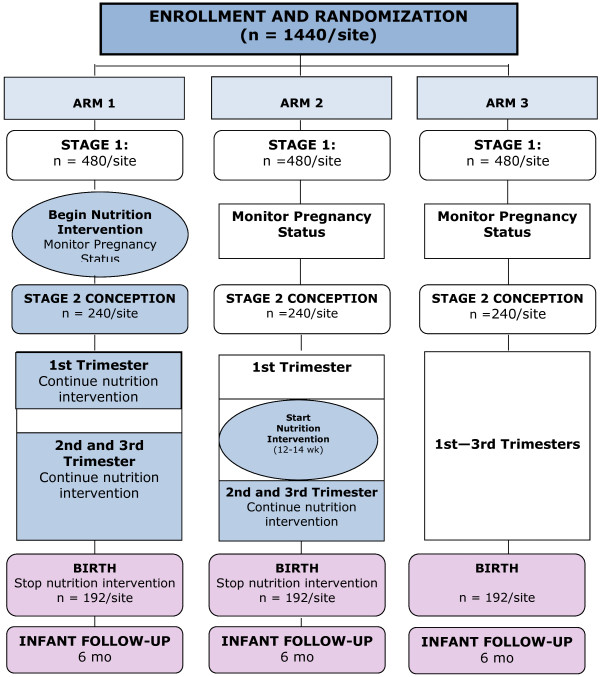
Consort diagram (subject numbers are for each independent site).

This is a 3-armed trial of a comprehensive nutrition supplement commencing at least 3 mo prior to pregnancy (Arm 1), at 12-14 wk gestation (Arm 2), or not at all (Arm 3). The trial is an individually randomized, longitudinal, non-masked, controlled trial. It will utilize the GN MNH Registry to facilitate identification of eligible subjects and will involve four sites in four different countries. A consort diagram for individual sites is given in Figure 1. A key feature of this trial is the difference in timing of starting the intervention, specifically at least 3 mo preconception (Arm 1) vs early 2nd trimester (Arm 2).

### Timeline (in months, commencing December 1, 2012)

0-12 Study preparations

13-18 Enrollment of 1440 eligible participants and baseline studies

38 Conception of 50% participants in each arm complete

47 All pregnancies completed

53 Maternal/infant follow-up completed (May 30, 2017)

60 Laboratory analyses, data analyses, reporting complete (Nov 30, 2017)

### Participants

Eligible participants will be identified primarily through the GN MNH Registry at each of the four sites.

Nulliparous women will be identified by site-specific strategies, including home surveys and marriage records. They will be randomized together with parous women, identified through the GN MNR, to one of the three arms.

### Inclusion criteria

16-35 y of age; parity 0-5; expectation to have first or additional pregnancy within next 2 y and without intent to utilize contraception.

### Exclusion criteria

Women with hemoglobin (Hb) ≤ 8 g/dL and nulliparous women who do not agree to hospital delivery (equipped for caesarian section) or/and do not have ready access to such a facility. Parous women who have had a previous history of pre-eclampsia or a history of prolonged labor associated with cephalopelvic disproportion will be excluded. Previous caesarian section will not necessarily exclude participation, a decision which will depend on reason for caesarian and intent to deliver where C-Section facilities are readily available.

### Ethical approval

Each participating research site has received ethical approval for the conduct of this trial through their local institutional review board (registered with US Office of Human Research Protection and with Federal Wide Assurances in place). The review boards provide initial review (with enhanced focus on local cultural practices and laws) and approve and conduct annual reviews of the trial to monitor enrollment, retention, and adverse events among other important issues. Importantly, each site has cultivated relationships with its Ministry of Health and community leaders in order to obtain input from communities and help ensure acceptance and effective implementation of community-based trials. All participants will provide written informed consent prior to participation.

### Study procedures

#### Enrollment

The optimal time post-partum for enrollment will vary by site; enrollment may start as early as 4 mo postpartum, but may be delayed until 8 mo depending on site-specific inter-pregnancy intervals. For nulliparous women, timing should be at least 4 mo before the estimated conception. Enrollment will occur after screening and informed consent in the home by the Home Visitor Research Assistant (HVRA) if inclusion criteria are met and there are no exclusionary criteria. Hb will be measured using blood from a finger stick and Hemocue™ instrumentation in all women. Enrollment of eligible women at each of the four participating sites will be undertaken over a 6 mo period. Prior to enrollment, potential participants will be advised that they will exit the trial if conception occurs before 3 mo of the biweekly home visits or if they are not among the first half of Stage 1 participants whose conception is confirmed after this first 3 mo period.

#### Randomization

Generation of the allocation sequence separately for each site has been undertaken by RTI International. A permuted block design with stratification by geographic cluster was used to generate the randomization sequence for assigning individual participants to a trial arm. The number of assignments to Arms 1, 2, and 3 were allocated with a ratio of 1:1:1 within blocks randomly varied in size between 3, 6, and 9. Stratification by cluster was used to ensure a similar number of participants randomized to each arm within cluster in order to provide geographic diversity across arms and for operational convenience given that the trial infrastructure in each country is organized around these clusters.

As each subject is enrolled, the responsible HVRA will contact the site data manager who will key the screening identification number into a computerized data management system (DMS) dedicated to the randomization process of enrollment.

Baseline data includes:

##### Questionnaires

Demographic information (including SES, water supply, sanitation, parental education and occupation); site-specific household food insecurity questionnaire [29] and indoor air pollution assessment. Questionnaires will be administered in the home by the local HVRA and will be completed within 1 wk of enrollment.

##### Medical history and anthropometry

A mobile assessment team will obtain past medical and obstetric history; anthropometric data from the mother including height and weight with calculation of body mass index (BMI); mid-upper arm circumference (MUAC); waist and hip circumference; and head circumference. A pregnancy test will be conducted to ensure that the participant is not pregnant. Paternal anthropometric measurements including height and weight will also be obtained at baseline whenever possible.

##### Biological sample collection

A phlebotomist will accompany the assessment team and collect a 0.5 mL venous or fingerstick blood sample from all willing participants in all three trial arms for Hb and dried blood spot collections.

##### Intervention

The nutrition intervention will be a multi-micronutrient (MMN) fortified lipid-based supplement (Nutriset, Malauney, France). In addition to the MMN and polyunsaturated lipids (linoleic 4.9 g and α-linolenic 0.59 g), the composition includes dried skimmed milk, soybean and peanut extract, sugar, maltodextrin stabilizers, and emulsifiers. The specific preparation is a modification of Nutributter by iLiNS based at UC Davis for research use with pregnant and lactating women (LNS P&L). This preparation is currently in use in studies in Ghana and Malawi where it has had formal acceptability testing. The micronutrient content of the daily 20 g supplement to be administered in this trial (Table 1) is unchanged from the LNS P&L except for an increase in the Vitamin D to conform to a recent update in the recommendation by the Institute of Medicine [30], and to allow for potentially lower bioactivity of the ergo-calciferol form chosen for acceptability to vegetarian populations. The zinc content was reduced to a level close to the recommendation of the Institute of Medicine for pregnant women [31]. The LNS P&L is water-free and has a long shelf life of 18 mo at room temperature in hot, humid environments.

**Table 1:** Nutrient content of lipid-based nutrition supplementation preparation (20 g daily portion)

**Nutrient**	**Amt**	**Nutrient**	**Amt**	**Nutrient**	**Amt**
Energy, *kcal*	118	Iron, *mg*	20	Thiamine (B1), *mg*	2.8
Protein, *g*	2.6	Magnesium, *mg*	65	Vitamin A, *μg*	800
Fat, *g*	10	Manganese, *mg*	2.6	Vitamin B12, *μg*	5.2
Linoleic acid, *g*	4.59	Niacin, *mg*	36	Vitamin B6, *mg*	3.8
α-Linolenic acid, *g*	0.59	Pantothenic acid (B5), *mg*	7	Vitamin C, *mg*	100
Calcium, *mg*	280	Phosphorous, *mg*	190	Vitamin D2, *IU*	1000
Copper, *mg*	4	Potassium, *mg*	200	Vitamin E, *mg*	20
Folate, *μg*	400	Riboflavin (B2), *mg*	2.8	Vitamin K, *μg*	45
Iodine, *μg*	250	Selenium *μg*	130	Zinc, *mg*	15

The intervention will be initiated in Arm 1 within 2 wk of enrollment to give time for baseline studies and in Arm 2 once participants in Stage 2 reach 12 wk gestation and have completed dietary assessments (if applicable), anthropometry measurements and biospecimen collection, and ultrasound exams. For each of these two arms, the intervention will be terminated at delivery.

Fourteen daily supplements will be provided biweekly in 20 g sachets with instructions to take one sachet per day. A rodent and water resistant plastic container will be provided to each participant for storage of the sachets. The supplement can be eaten as is or mixed in with other foods depending on the preference of the participant. A record will be maintained indicating if sachet is taken alone or with meals. Instructions will be given to the participant that although the supplement can be added to other foods before eating, it should not be added to foods while cooking.

#### Follow-up procedures: stage 1 (Preconception)

Biweekly home visits and assessment for all three arms (more frequently for first 2 wk or for longer period if issues with compliance) will commence within 2 wk of enrollment. Menstrual history will be obtained at each visit and a urine pregnancy test will be performed if last menstrual period (LMP) was > 4 wk prior or mother thinks she might be pregnant. Pregnancy testing will also be undertaken at every visit after the first 3 mo postpartum if menses have not yet resumed or are irregular. Maternal morbidity will be monitored at each visit for all participants. A calendar will be provided to the women and they will be trained to record history of morbidity and menses on a daily basis. Discussion will include plans for prenatal care, location of delivery, and exclusive breastfeeding support (late 3rd trimester onwards). Women in Arm 1 will be weighed monthly and BMI calculated by the Field Supervisor. The HVRAs will be supported wherever possible by local community volunteers or/and health workers (especially in India and by nurses in the widespread DRC communities).

Compliance of supplement use will be monitored by self-reported history and sachet collection during biweekly visits. Women will also mark off daily supplement compliance on the calendar noted above. Compliance will also include random independent audits by other research personnel.

Results of monthly weight checks for Arm 1 participants will identify those with BMI ≤ 20 initially or at any subsequent stage and those participants will receive an additional balanced protein/energy (300 kcal/d; 12% calories from protein and no added micronutrients; Nutriset, Maluaney, France) supplement, and weight will be monitored monthly. Once initiated, the second supplement will be continued until the end of the trial or the completion of pregnancy, whichever is the soonest.

Participants on the intervention will be cautioned not to take other micronutrient supplements or fortified supplemental foods while on the trial intervention. Use of additional supplements will be monitored and recorded at the monthly assessment visits conducted by the assessment team.

#### Follow up procedures: stage 2 (Pregnancy)

The first 240 participants in each arm at each site to conceive after 3 mo will enter Stage 2 (participants must have received at least 3 mo nutrition intervention/home visits). The remaining participants in Stage 1 will discontinue the trial. An exit visit will include Hb measurement (Hemocue™) and appropriate referral as necessary for follow-up care.

*Biweekly visits* by the HVRA will continue as before for pregnant participants retained in the trial. The HVRA will encourage participants to have prenatal care and hospital delivery. The HVRA or supervisor will also be in contact with the individual/facility responsible for delivery and this will be on a longitudinal basis in the case of community birth attendants and home deliveries. Participants in Arm 2 will be advised to stop taking iron and folate supplements once they start the nutrition intervention at 12 wk gestation. Participants in all arms will be advised at 12-14 wk gestation to commence prenatal care, which, in the case of Arm 3, may include supplements provided by caregiver.

During pregnancy, monthly weight checks will continue for Arm 1 and be initiated for Arm 2 at the time the nutrition supplement is started. Extra energy/protein supplement (see above) will be provided for women in Arms 1 and 2 if 2nd and 3rd trimester pregnancy weight gain fails to reach recommended guidelines (approximately 2 kg/mo for underweight and normal weight women and 1 kg/mo for overweight and obese women).

##### Dietary assessment

Half of the trial women in both Arm 1 and Arm 2 will be selected for a dietary assessment at the biweekly visit when pregnancy is confirmed. A research nutritionist will obtain two multiple-pass 24-h dietary recalls (2-4 wk apart) from every other participant in Arms 1 and 2 prior to 12 wk gestation (i.e. 120/arm, 240/site). Both 24-h dietary recalls will be conducted in the participant’s home.

##### Assessment visits

At 12 and 34-36 wk gestation, maternal anthropometry will be conducted for women in all arms and Hb will be repeated (with referral for care if Hb <8 g/dL). Biological samples, including maternal blood (30 mL), spot urine, and stool will be conducted for women in Arms 1 and 2. If the participant declines to provide a venous blood sample, research personnel will request an optional simple finger stick for blood spot collections. Methods for processing and storage while maintaining cold chain will be carefully adhered to. HVRA will also accompany participants in all arms to the health center or district hospital for fetal ultrasounds at 12 and 34-36 wk gestation, organizing transport as necessary. Only after completion of the 12 wk gestation assessment visit will Arm 2 commence the same nutrition supplement as Arm 1. Arm 3 will continue to receive biweekly visits monitoring maternal infectious disease morbidity and MUAC.

*Monthly weight checks* will continue and extra energy/protein will be provided to women in Arms 1 and 2 who do not maintain recommended weight gain during pregnancy (see above). Pregnancy complications will be recorded as adverse events. These include miscarriages and still births, with estimation of gestational age of latter and weight when feasible.

#### Follow up procedures: stage 2 (Delivery)

For women in all arms, deliveries will whenever possible be in local birth facilities/regional hospitals. Whenever trained hospital personnel are at the delivery and mother provides consent, they will collect amnion and, after delayed cord clamping, cord blood. Maternal blood, stool sample and vaginal swab will also be collected. Infant birth length [32] and weight will also be obtained from all participants within 12 h of delivery. This applies to home as well as hospital deliveries and a team will be on call at weekends for the 12 h measurements.

Nutrition supplements will be discontinued at time of delivery for Arms 1 and 2.

#### Follow up procedures: stage 2 (Postpartum)

*Biweekly visits* by the HVRA will continue as before. HVRA will record all severe maternal morbidity and infant morbidity requiring health center/hospital visits admission for malaria, unspecified fever, respiratory disease, or diarrhea. Morbidity cared for at home will also be recorded with assistance of initial participant education and calendar. The project coordinator will be alerted in case of neonatal/infant mortality and need for verbal autopsy. For all of these duties, HVRAs will be supported and monitored by field supervisors. The HVRAs will also check on details of infant feeding at each visit, supporting exclusive breast feeding; noting any other fluids given with details and also when first weaning foods are offered if before 6 mo.

*Assessment visits* will be conducted at 14-d, 1, 3, and 6 mo of age to collect neonatal and infant anthropometry for offspring of women in all arms by members of the Assessment Team. Maternal weight will also be measured at 6 mo visit. An optional blood sample will be collected at 14 d and at 3 mo postpartum for women in Arms 1 and 2 and at 3 mo postpartum in Arm 3. For infants in all trial arms, optional blood collection will be limited to a fingerstick at 2 wk and at 3 mo of age, with parental consent. From previous experience it is estimated that 50% of subjects will consent to blood collections. The HVRA will assist the participant in collecting 20 mL hand expressed breast milk at 14 d and 3 mo postpartum. All samples will be collected, stored, processed, and transported maintaining cold chain.

A summary of study procedures for each participant is shown in Tables 2, 3 and 4.

**Table 2:** Summary of study procedures for Stage 1 of participation

**Stage 1: Preconception**	**Week of participation**		
	**0**	**1**	**2 until conception**
**Screening/Consent**	X		
Enrollment/Randomization*	X		
**Baseline data collection**		X	
*(SES, environmental, maternal and paternal anthropometry, health hx)*			
**Biweekly home visits** (begin within 2 wk of enrollment)			
*Monitor menses/confirm pregnancy*			→ → →
*Primary nutrition intervention (Arm 1)*			→ → →
*Energy/Protein supplement (prn, Arm 1)*			→ → →
*Maternal Morbidity*			→ → →
*MUAC*			→ → →
**Maternal monthly weight** (Arm 1 only)			→ → →
**Maternal blood sample collection** (all arms)		X	

*Nulliparous women will be enrolled at a point in time estimated to be at least 4 mo before first pregnancy occurs.

**Table 3:** Summary of study procedures for Stage 2 (pregnancy) of participation (timeline begins with conception)

**Stage 2: Pregnancy & Delivery**	**Stage of pregnancy (wk)**			**Delivery**
	**0-12**	**13-24**	**25-birth**	
**Biweekly home visits**				
*Nutrition intervention*				
*Arm 1*	→ → → → →			
*Arm 2*	→ → →			
*Kcal/Pro supplement (prn, Arms 1 & 2) prn*	→ → → → →			
*Maternal morbidity*	→ → → → →			X
*MUAC*	→ → → → →			
**Dietary assessment** (two, 24-hr recalls in every other participant in Arms 1 & 2)	XX			
**Maternal monthly weight**	XXX	XXX	XXX	
	(Arm 1)	(Arms 1 & 2)	(Arms 1 & 2)	
**Fetal ultrasound**	X (12 wk)		X (34-36wk)	
**Maternal biologic sample collection**	X (12 wk)		X (34-36 wk)	X
	(Arms 1 & 2)		(Arms 1 & 2)	
**Maternal anthropometry and vitamin/mineral use**	X (12 wk)		X (34-36 wk)	
**Infant birth length & weight**				X

Procedures are for all women in all arms unless otherwise noted.

**Table 4:** Summary of study procedures for Stage 2 (postpartum) of participation (timeline begins at delivery)

**Stage 2: Postpartum/infant**	**Time postpartum**				
	**12 hr**	**14 d**	**1 mo**	**3 mo**	**6 mo**
**Neonatal/Infant anthropometry**	X	X	X	X	X
**Maternal anthropometry**					X
**Maternal morbidity** (biweekly)	→ → → → → →				
**Infant morbidity** (biweekly)	→ → → → → →				
**Breast milk collection**		X		X	
**Maternal biological sample collection**		X		X	
		(Arms 1&2)			
**Infant biological sample collection**		X		X	

### Outcome measures

#### Primary outcome: birth length-for-age Z-score

Highly trained Research Assistants (Assessment Team) will obtain these early neonatal length measurements at 12 h of age (neonatal stadiometer, Ellard Instrumentation, Ltd, Monroe, WA), with skills that include gentle solicitation of a Babinski response to achieve feet at right angles to the lower limbs [32].

Other anthropometry measurements obtained at this time include infant weight (electronic scales sensitive to 10 g), head circumference (plasticized measuring tape accurate to 1 mm), and MUAC with data being converted to Z-scores automatically by the computer on data entry.

### Secondary outcomes

1) Infant LAZ at age 0.5, 1, 3 and 6 mo postnatal

Research assistants (Assessment Teams) will obtain infant anthropometry at age 0.5, 1, 3, and 6 mo of age. Measurements will be converted to Z-scores using the current WHO growth standards.

2) Estimate longitudinal fetal growth

Ultrasound measurements will be undertaken at 12 and 34-36 wk gestation with the goals of confirming gestational age (use ultrasound estimate if >7 d different from LMP estimate) and estimating longitudinal fetal growth, the latter from repeated measurements of crown-rump, femur and humeral length. Biparietal diameter and abdominal circumference will also be measured. Measurements will be conducted by specially trained ultrasonographers or obstetricians. Ultrasound reading will be reviewed by local site expert(s) and a random sample will be reviewed by a single expert to assure quality control.

3) Mean birth weight and incidence of LBW infants

Incidence of LBW is a recognized risk factor for mortality and the other peri- and neonatal outcomes targeted in this proposal, as well as for later non-communicable disease. Birth weight will be recorded at delivery.

4) Perinatal mortality

Pregnancy losses >20 wk gestation will be recorded as stillbirths, together with intrapartum losses, maternal ‘near deaths’ and neonatal deaths up to 1 mo of age as well as early infancy deaths from 1-6 mo of age. Verbal autopsy results for all neonatal and early infant deaths will be obtained from the GN MNH Registry [26].

5) Incidence of severe neonatal and infant infectious disease

Outcome measure is number of acute visits/admissions to health center/hospital for severe infectious disease. Diagnosis and treatment will also be recorded. It is further intended to collect minor morbidity data. These data will be obtained with use of a trial calendar with appropriate training on use and collected at biweekly home visits. Infant vaccination history will be recorded at the assessment visit occurring at 6 mo of age.

### Laboratory outcome measures

Activities Common to Secondary Outcomes 6-9.

6) Epigenome of mother, fetus and offspring

Maternal environmentally-induced improvement in her epigenome provide an appealing hypothesized mechanism by which this preconception nutrition intervention is responsible, at least in part, for the hypothesized resulting phenotypic improvements in her offspring including pre- and post-natal growth. In particular it is hypothesized that DNA methylation patterns in global and gene specific studies will vary with a maternal nutrition intervention commencing pre-conception vs. 12-14 wk gestation vs. no maternal nutrition supplement. Patterns will be compared in DNA from maternal blood (longitudinal); fetal tissue (amnion); cord blood and infant blood at 3 mo post-partum. Maternal samples will be collected at baseline (preconception), 12 and 34 wk gestation; delivery, 2 wk and 3 mo postpartum. Infant finger stick blood will be collected at 2 wk and 3 mo.

For blood samples, 90 μL of venous blood will be applied to each circle (× 4) of dried blood spot cards (Whatman 903 Protein Saver). The card will be protected in a clean environment out of direct sunlight until dry. Ten cards will be sealed in a multi-barrier pouch containing desiccant packs and monitored for humidity on a weekly basis using humidity indicator cards. The pouches will be stored at room temperature until laboratory analyses. Sections of amnion will be placed immediately in cryovials and subsequently stored at−80C. Samples will be shipped in part to UCD where procedures will include DNA extraction, Chip-Seq Library preparation and Illumina 450 k methylation analyses.

7) Deep phenotyping of metabolic and nutritional status

Enhanced inflammation and oxidative stress represent a “final common pathway” to intrauterine growth retardation and are potentially mediated by a number of different stimuli, including maternal diet and nutritional status (e.g. energy inadequacy, micronutrient deficiencies, fatty acid imbalance), insulin resistance, poor water quality, recurrent infectious stimuli, indoor air pollution, and maternal stress (“allostatic load”). The effects of chronic inflammation and oxidative stress have potential to alter pregnancy outcomes, including the fetal and postnatal growth potential, nutrient utilization and nutritional status, and immunologic development and function of mother and of the offspring. The proposed LNS intervention, through anti-inflammatory and antioxidant features, may beneficially alter the maternal metabolic profile, especially for those who initiate the LNS preconception.

The outcome represents deep phenotyping by measuring in maternal tissues: hormones, metabolites, measures of inflammation, oxidant stress and immune function/status, and nutrient biomarkers as possible indices of fundamental metabolic alterations resulting from improved long-term maternal nutrition in food insecure populations. The potential contributions of these assays include early prediction of impaired fetal growth and possibly enhanced understanding of the mechanisms responsible for growth improvements in offspring of poor women provided with food supplements.

• Cold chain and cold sample storage facilities will be obtained/upgraded as necessary at all community hospital sites (except DRC);

• Specialized training of research personnel to ensure proper sample collection, transportation and storage. Vigorous training of birth attendants and other hospital personnel will be undertaken for collection/freezing of cord blood and amnion samples;

• Site-specific arrangements will be made for optimal transport of frozen samples while maintaining cold chain to UCD for laboratory analyses. Jawaharlal Nehru Medical College (JNMC) will undertake laboratory analyses on site to the greatest extent possible.

**Figure 2:**
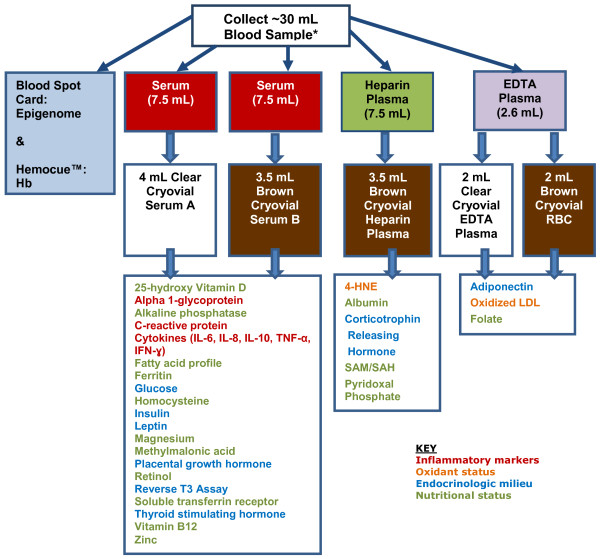
Blood collection and analyses for maternal longitudinal samples.

Biological samples (blood, urine, fecal) will be collected from all maternal participants who consent at delivery, and at 14 d and 3 mo postpartum (Figure 2); additional samples will be collected at 12 and 34-36 wk gestation in Arms 1 and 2. The following assays will be performed:

• Maternal systemic and/or gastrointestinal inflammatory markers: hsCRP, AGP, cytokines (IL-6, IL-8, IL-10, TNF-α, IFN-γ); urine and fecal neopterin, and fecal calprotectin, alpha-1-antitrypsin and myeloperoxidase.

• Maternal oxidant status [33] will be assessed by measuring oxidized-LDL and 4-HNE (4-hydroxynonenal, produced during oxidative stress and subsequent lipid peroxidation of polyunsaturated fatty acids).

• Maternal endocrinologic milieu will be assessed by biomarkers of placental function: placental growth hormone [34]; indices of iodine and energy utilization: thyroid hormone assays (TSH and Reverse T3), serum glucose, insulin (and calculated HOMA-IR: (fasting insulin [mLU/L]× fasting glucose (mmol/l]/22/5)); leptin and adiponectin [35]; and maternal stress (“allostatic load”): serum corticotrophin releasing hormone (CRH).

• Maternal nutritional status will be assessed with the following biomarkers: iron and zinc status (serum ferritin, AGP, soluble transferrin receptor, zinc); essential fatty acid profile; Vitamin B12 and methylmalonic acid; 25-hydroxy Vitamin D; RBC folate; retinol; pyridoxal phosphate, SAM/SAH, homocysteine; alkaline phosphatase; albumin; magnesium.

Infant finger stick samples will be utilized for epigenetic studies as first priority. Any residual blood will be used for modified phenotyping. Infant stool samples will also be analyzed for biomarkers of gut inflammation including calprotectin, neopterin, alpha-1-antitrypsin and myeloperoxidase.

8) Microbiome

The maternal intestinal microbiome will be evaluated at 12 and 34-36 wk gestation and at delivery. A partial stool sample will be collected at each time point with the addition of a vaginal swab at delivery. The contribution of this outcome is to provide insight into the role of nutrition from conception on the nature of the microbiome with all of the now recognized implications of the latter for the development of the host immune system and nutritional status [36]. The microbiome will be characterized by next-generation DNA sequencing assays.

Partial fecal samples will be collected at 14 d and 3 mo to characterize the infant microbiome (as above). As with other samples, the fecal samples for microbiome will be used to compare with maternal microbiome data.

9) Composition of breast milk

We hypothesize that improved maternal nutrition at the time of greatest plasticity in early pregnancy will favorably influence maternal metabolic and nutritional status throughout pregnancy and thus potentially the composition of breast milk in terms of hormonal content, immune factors, cytokines, and gut growth factors. Emerging data in the literature support the impact of maternal metabolic status on these bioactive factors [37]. The effects these differences may have on early postnatal growth have not been systematically examined.

This outcome will determine differences between the three arms in composition of breast milk at 14 d and 3 mo postpartum, with focus on potential growth factors, inflammatory mediators, hormones and adipokines, markers of maternal oxidant stress and antioxidant capacity of milk, all of which may directly or indirectly influence infant growth and development.

Bioactive components of human milk for analyses:

• Nutrient composition: fatty acid profile; macronutrients (lactose, protein, lipids, glucose); selenium; iodine; Vitamin B12; folate; and Vitamin B6

• Growth factors (somatic and intestinal/mucosal development and immunologic protection): IGF-1, EGF (epidermal growth factor), TGF-α and TGF- β

• Cytokines: (IL-6, IL-8, IL-10, TNF-α, IFN-γ), and hsCRP

• Hormones and adipokines: insulin, leptin, and adiponectin

• Oxidative stress: F2-Isoprostanes, and 4-HNE

• Antioxidant capacity: total antioxidant capacity (TAC), ferric reducing ability of plasma (or milk) (FRAP)

### Data management & statistics

#### Trial power and sample size

Sample size was determined to test the primary hypothesis based on the primary outcome of LAZ at birth with 80% power at each individual site, while maintaining an overall trial-wise Type I error rate of 0.05 across all planned primary hypothesis tests (two tests comparing Arm 1 vs Arm 2 and Arm 1 vs Arm 3 at each of four sites). Thus an alpha level of 0.00625 was specified to account for the 8 planned primary comparisons. Assuming an alpha level of 0.00625, a 2-sided test, and a standard deviation of 1.0 for the primary outcome, LAZ, 192 evaluable women per arm at each site will allow detection of an effect size of 0.37 with 80% power. Accounting for 20% attrition during pregnancy requires that 240 women per arm enter Stage 2 at each site, and assuming that 50% of women randomized at Stage 1 will get pregnant and move to Stage 2 requires 480 women per arm enrolled at each site. Keeping the number entering Stage 2 at 240 per arm, if attrition during pregnancy is 10% instead of 20%, 216 evaluable women per arm will allow detection of an effect size of 0.35.

If we consider only the Arm 1 vs Arm 2 comparison as primary, given 192 evaluable women per arm at each site for a total of 768 women per arm over all four sites, setting an alpha level of 0.025 for each primary hypothesis test will allow detection of an effect size of 0.18 with 90% power or an effect size of 0.20 with 95% power for each overall comparison. When data are combined for identical trials undertaken in all four participating countries, power will be sufficient to demonstrate a significant reduction in incidence of LBW between Arms 1 and 3 with a trend for decreasing perinatal mortality.

#### Adverse events

Adverse events will be monitored continuously on an ongoing basis by recording on a data form any of the following situations:

• Any fatal or life threatening event occurs to the participating woman, fetus or infant, or

• Any serious and unexpected adverse event occurs

The specific adverse events monitored in this trial include early termination of pregnancy, adverse pregnancy outcome, adverse neonatal event, hospitalization (mother or child) due to acute illness, allergic reactions, diarrhea, and vomiting. All such events, including other unexpected events will trigger completion of the adverse events form, and will be reported to the site PI, the Data Coordinating Center and the overall trial PI within 48 h for all deaths and within 7 d for other adverse events.

#### Interim data monitoring and analyses

The Global Network Data Monitoring Committee (DMC), designated by NICHD, will monitor the safety of the trial. The DMC will monitor the trial for safety at approximately 6-m intervals, once all countries have commenced recruitment. The adverse events enumerated above, as well as other unexpected serious adverse events will be tabulated by treatment group for the DMC to review. While the DMC report will be masked to treatment group (designated only as groups A, B and C) for routine monitoring, the DMC may be unmasked to comprehensively review the safety profile for the trial participants.

The trial has no planned interim efficacy looks or relevant stopping rules because the primary outcome is only available at birth, with a large lag between recruitment/randomization and availability of the primary outcome (at least 12 mo), and a number of important secondary outcomes are assessed at 6 mo of age.

#### Statistical analyses

This trial is designed to formally test mean differences in the primary outcome of birth LAZ between women randomized to receive a daily nutrition supplement beginning 3 or more months prior to conception (Arm 1) and women randomized to receive the same daily nutrition supplement beginning at 12 wk gestation (Arm 2), and between Arm 1 and a control group of women (Arm 3) who will not receive the nutrition supplement. Analyses will be conducted separately by research site. In a secondary analysis outcomes will be compared across all four sites using the combined data. Following an intent-to-treat approach for all offspring who have a primary outcome determined, individuals will be analyzed in the intervention group to which they are assigned regardless of intervention received. The primary analysis will compare mean LAZ between groups using a linear model with LAZ as the outcome and intervention group as the primary predictor to test the two primary hypotheses, namely that: (1) mean LAZ at birth differs between Arm 1 and Arm 2, and (2) mean LAZ at birth differs between Arm 1 and Arm 3. First, the two degree of freedom F-test will be used to test the hypothesis that the mean LAZ in at least one arm differs from the other two versus the null hypothesis that all three groups have equal mean LAZ. If that test is significant at the 0.0125 level then the two primary hypotheses will be tested at the 0.00625 level. Secondary analyses using multivariable regression models may adjust for the randomization cluster and any other critical covariates or confounders that may be imbalanced across the treatment groups (see below). The same modeling approach will be used to evaluate the trial hypotheses over all sites combined, while controlling for research site.

Since the primary outcome of mean LAZ will only be available on a subset of those randomized (women who get pregnant and deliver a live birth, with LAZ evaluated), we will ensure that we still have balance across the three treatment groups for key covariates and confounders for the group with the primary outcome available. These will include factors such as maternal BMI, maternal age, parity, and a summary measure for baseline socio economic status. If there is any suggestion of imbalance on any of these covariates across the three treatment groups (indicated by p < 0.1), such covariate(s) will be adjusted for in the primary analysis for the primary and all secondary outcomes.

In addition, as a further check to ensure that evaluating only a subset of those randomized is not skewing the results in any unexpected manner, we will construct a composite binary secondary outcome that will be evaluated alongside the primary outcome on all randomized women who get pregnant. We provisionally define this outcome as live birth free of growth failure, with the latter defined as LAZ < −2 SD at or within 12 h of birth. This and other binary outcomes (such as LBW and perinatal mortality) will be analyzed using chi-square tests (unadjusted) or robust Poisson regression (adjusted for cluster and/or other covariates that may be imbalanced) to produce estimates of relative risk [36].

Secondary outcomes evaluated will include infant growth over the first 6 mo of life, prevalence of LBW, and prevalence of perinatal mortality (stillbirths and neonatal deaths up to 1 mo of age). Infant growth will be examined based on LAZ at birth, 0.5, 1, 3, and 6 mo with linear repeated measures models using generalized estimating equation methods with robust variance estimators to account for the correlation of LAZ across time within individual infants. Models will include terms for intervention group, time and the group by time interaction to evaluate whether the three groups differ in changes in mean LAZ across time. For the longitudinal analyses, missing outcome measures will be treated as missing at random. Other secondary outcomes will be evaluated using model-based approaches as applicable. The models will allow for inclusion of covariates, such as socio-economic status, maternal age, or maternal smoking, to account for potentially confounding effects of baseline differences across groups. All models using data combined over the four sites will control for research site.

Covariates that occur after randomization will not be included in the primary models assessing intervention group differences with respect to the primary outcome, LAZ at birth, or secondary outcomes including infant growth in the first 6 mo as these covariates may in theory be affected by the intervention and lie in the causal pathway between intervention and growth. However, in subsequent analyses such as mediation analyses, factors known to affect infant growth may be explored for direct and indirect effects on outcomes.

## Discussion

### Innovation & significance

Key innovative features of this maternal nutrition intervention project are [1] the longitudinal trial commencing prior to conception, with, importantly, a range of intervention intervals before conception (as birth size is related to inter-pregnancy intervals [38]); [2] the primary focus on length, but with a wide range of public health and laboratory outcomes; [3] the special emphasis on intervention-related changes in the epigenome of mother, fetus and infant; and [4] the relatively large predicted effect size for the primary outcome of newborn LAZ compared with that observed for maternal nutrition interventions commencing when gestation is well established.

### Sustainability

Assuming that the primary hypothesis is correct, an important initial aspect of sustainability will be to ascertain the longer term benefits of this trial. This project covers the first half of the ‘First 1000 Days.’ Assuming the hypothesized benefits to fetal growth are confirmed, especially by enhanced early neonatal length, it will be of outstanding importance to know the extent to which beneficial outcomes of this preconception intervention model are carried through to the second half of the ‘First 1000 Days’. Positive birth/early neonatal results will give time to prepare economically and organizationally to undertake anthropometry and tests of cognitive development from 6 mo of age into early childhood.

Longer term, positive results of this project will make a powerful case for shifting current paradigms on prevention of retarded linear growth in the ‘First 1000 Days’ and its life-long adverse associations. The case will be strengthened by the multi-country, multi-continent, multi-cultural organization of this project which could, therefore, without essential further confirmation, result in adjusted strategies and recommendations. The promotion of optimal preconception maternal diets by the world’s major health organizations will be especially vital because of the implications for prioritizing promotion of optimal nutrition for all women of child-bearing age (including adolescent girls),which is a challenging task.

In addition to favorable anthropometric outcomes, encouragement to pursue measures for sustainability may result from the outcomes of the associated laboratory research, for example, evidence of reversing unfavorable epigenomic patterns by preconception nutrition intervention with subsequent transmission of these favorable results to the fetus.

### Risks

This project is very low risk. The theoretical small risk of increasing the frequency of cephalo-pelvic disproportion is being minimized by giving priority to inclusion of participants who have had a previous uncomplicated vaginal delivery and on deliveries in facilities that have caesarian section capacity. Nulliparous women will not be excluded provided delivery is in a facility equipped for Caesarian sections. A second potential risk is increasing prevalence of overweight/obesity in developing countries, including women of child-bearing age. These women and their offspring may still benefit from micronutrient supplements and, to minimize this risk, will not be candidates for additional calorie-protein supplements.

### Trial organization and management

#### Management plan for this project

**Figure 3:**
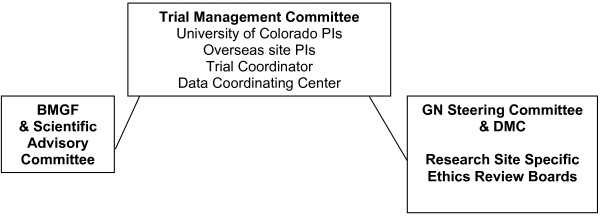
Organizational chart for month-to-month supervision of project.

Overall oversight of the project and leadership is provided by the Nutrition Program of the Bill & Melinda Gates Foundation together with the International Scientific Advisory Committee formed specifically for this project and with the NICHD Global Network for Women’s and Children’s Health Research (Steering Committee and DMC) (Figure 3).

#### List of participating research sites for this project

• University of Colorado Denver, Aurora, CO: Drs. Hambidge and Krebs provide overall trial management and oversight with support of J Westcott, including fiscal management.

• Institute for Multidisciplinary Health (IMSALUD), Guatemala City, Guatemala: Dr. Ana Garces provides the in-country leadership for the Guatemala site.

• Aga Khan University (AKU), Karachi, Pakistan: Dr. Omrana Pasha provides in-country leadership for the Pakistan sites. AKU collaborates with Dr. Robert Goldenberg, Columbia University, for the Global Network, who will serve as an advisor and interface with the Global Network.

• Jawaharlal Nehru Medical College (JNMC), Belgaum, Karnataka, India: Drs. Bhala Kodkany and Shiva Goudar provide in-country leadership of the trial. The JNMC site collaborates with Dr. Richard Derman, Christiana Health Care, for the Global Network.

• Kinshasa School of Public Health (KSPH), Kinshasa, Democratic Republic of the Congo (DRC): Dr. Antoinette Tshefu serves as the lead of the DRC site. Dr. Carl Bose, University of North Carolina, serves as the Global Network partner of the DRC site.

• RTI International, Durham, NC: Dr. Elizabeth McClure serves as the director of the data center activities at RTI for this project and the Global Network, supported by Kristen Stolka and by Dr. Abhik Das, the senior statistician of the preconception trial.

**Figure 4:**
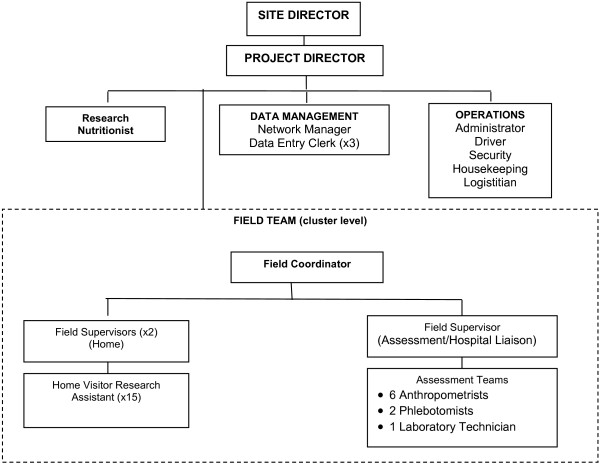
Organization of individual research sites.

Each research site has a cadre of trained research personnel including physicians, nurses, research assistants, nutritionists, traditional birth attendants or equivalent, and laboratory technicians, who are trained in data and sample collection, and data managers who oversee the information technology, data entry and data edits. Generally, the site organization is described in Figure 4.

## Abbreviations

AKU: Aga Khan University; BMGH: Bill and Melinda Gates Foundation; BMI: Body mass index; DMC: Data Monitoring Committee; DRC: Democratic Republic of the Congo; GN: Global Network; Hb: Hemoglobin; HVRA: Home Visitor Research Assistant; IMSALUD: Multidisciplinary Institute for Health; IUGR: Intrauterine growth retardation; JNMC: Jawaharlal Nehru Medical College; KSPH: Kinshasa School of Public Health; LAZ: Length-for-age Z-Score; LBW: Low birth weight; LMP: Last menstrual period; MNH: Maternal Newborn Health; MMN: Multi-micronutrient; MUAC: Mid-upper arm circumference; NICHD: National Institutes for Child Health and Human Development; PRN: As situation requires; PTB: Preterm birth; RTI: Research Triangle Institute, International; UCD: University of Colorado Denver; WAZ: Weight-for-Age Z-Score.

## Competing interests

The authors declare that they have no competing interests.

## Authors’ contributions

KMH conceived of the trial, and was supported in the design by NFK. JEW contributed to finalizing details of the project and manuscript. The overseas Site Directors, SG, OP, AT &, especially, AG all contributed to the development of the trial. RLL is responsible for the dietary component, DF for the microbiome, and JF for the epigenetic studies. Other members of the Global Network Steering Committee including LLW, MKT, RD, BK, CB and RG, participated in critiquing early stages in the evolution of this project. EM provided vital strategic advice. KS has contributed to the development of the final protocol and she and JEW have leading roles in data management and trial implementation/monitoring. Both AD & DW were primarily responsible for the power testing/statistical sections. SS has primary responsibility for the initiation and monitoring of this trial. Additional members of the Women First: Preconception Nutrition Trial group include the project coordinators at each of the four overseas sites (Lester Figueroa (Guatemala), Sangappa Dhaded (India), Adrien Lokangaka & Dieudonné Bidashimwa (DRC), and Sumera Ali (Pakistan); Rebecca L Lander (UCD); Linda L Wright (NICHD); and Dennis Wallace (RTI). All authors read and approved the final manuscript.

## Pre-publication history

The pre-publication history for this paper can be accessed here:

http://www.biomedcentral.com/1471-2393/14/111/prepub

## Supplementary Material

Additional file 1 Women First Scientific Advisory Committee. Click here for file
