# Supplementary material for: Preconception maternal nutrition: a multi-site randomized controlled trial
Source: BMC Pregnancy Childbirth. 2014 Mar 20;14:111. doi: 10.1186/1471-2393-14-111 (PMC4000057; doi:10.1186/1471-2393-14-111)
Supplement: Additional file 1 — Women First Scientific Advisory Committee. [file 1471-2393-14-111-S1.doc]

**Women First Scientific Advisory Committee**

**May 2013**

**Laura Caulfield, PhD**

Bloomberg School of Public Health

Johns Hopkin University

615 North Wolfe St., W2041

Baltimore, MD 21205

Email: lcaulfie@jhsph.edu

Phone: 410-955-2786

Fax: 410-955-0196

**Katherine Dewey, PhD**

Director, Program in International & Community Nutrition

Distinguished Professor, Department of Nutrition

University of California, Davis
3253 Meyer Hall
One Shields Avenue
Davis, CA 95616

Email: kgdewey@ucdavis.edu

Phone: (530) 752-1992

Fax: (530) 752-3406

**Caroline Fall, BSc, MBBChir, FRCP, FRCPCH**

Professor of International Epidemiology
Faculty of Medicine
University of Southampton
Southampton General Hospital
Mailpoint 801, South Academic Block
Tremona Road
Southampton, UK SO16 6YD

Email: [chdf@mrc.soton.ac.uk](mailto:chdf@mrc.soton.ac.uk)

**Stephen Kennedy, FRCOG**

Chair, Nuffield Department of Obstetrics & Gynaecology

Director, Intergrowth-Diet Project
University of Oxford
Level 3, Women's Centre,

John Radcliffe Hospital
Oxford, UK OX3 9DU
Email: [stephen.kennedy@obs-gyn.ox.ac.uk](mailto:stephen.kennedy@obs-gyn.ox.ac.uk)

Phone: 01865 221004
Fax: 01865 769141

**Caroline Relton, BSC, PhD**

Professor of Genetic & Epigenetic Epidemiology

Institute of Genetic Medicine

Newcastle University

Phone: +44 (0) 191 2418623

Fax +44 (0)191 2418666

Email: [caroline.relton@ncl.ac.uk](mailto:caroline.relton@ncl.ac.uk)

and

School of Social & Community Medicine

University of Bristol

Email: [caroline.relton@bristol.ac.uk](mailto:caroline.relton@bristol.ac.uk)

Tel. +44 (0)117 3310072

Fax +44 (0)117 3310123

**Beth McClure, PhD**

Senior Research Statistician

RTI International
3040 East Cornwallis Road
Post Office Box 12194
Research Triangle Park, NC 27709-2194
Email: [Mcclure@rti.org](mailto:Mcclure@rti.org)

Phone: 919-541-6000

***Ex Officio Members***

**Shelly Sundberg, PhD**

Senior Program Officer

Family Health Division

Bill and Melinda Gates Foundation

PO Box 23350

Seattle, WA 98102

Email: [shelly.sundberg@gatesfoundation.org](mailto:shelly.sundberg@gatesfoundation.org)

Phone: (206) 770-1846

**Caroline Signore, MD, PhD**

Deputy Director, Division of Extramural Research

*Eunice Kennedy Shriver* National Institute of Child Health and Human Development (NICHD)

6100 Executive Blvd Room 4A05C, MSC 7510
Bethesda Md 20892-7510
Email: signorec@mail.nih.gov

Phone: 301-496-5577
